# Medical Opinion and Movement

**Published:** 1909-01-09

**Authors:** 


### Medical Opinion and Movement.

At a meeting of the Académie des Sciences Dr. E. de Bourgade la Dardye reported on a method he has devised by which he seeks to penetrate the deep tissues with the Röntgen rays. He points out that the destructive action of the rays is not effective on growths in the deep tissues, owing to the greater part of the rays being absorbed by the skin and subcutaneous tissue. His idea is that by injecting sulphide of zinc into the neoplasm, a short exposure to the rays causes absorption of the radiations by this chemical, which then becomes, by its phosphorescence, an active centre of considerable duration. The author states that in this way he has been able successfully to destroy several tumours, and gives particular details of two cases, one of lupus of the nose and the other of tuberculosis of both testicles. In the first case the zinc sulphide was injected into the nostrils, the nose was exposed to the rays for ten minutes, and the salt thus "activated" was kept in position for about twelve hours. After the fourth application the internal vegetations had completely disappeared and the condition was cured. In the second case the zinc sulphide was injected through the fistulæ of the tumour and the *x*-rays were applied for five minutes every second day. At the end of six weeks the improvement was such that the patient was able to return to his work.

Seven years ago Dr. A. Fränkel drew attention to a somewhat rare and acute morbid condition of the lungs, which he termed "bronchiolitis obliterans," and since then two or three further cases have been reported by other authors. At a recent meeting of the Berlin Society of Medicine Dr. Fränkel made a report on some further cases observed by him. The causative agent of the disease appears to enter the lungs with the inspired air. The subjects of the disease are generally young and workers in metals, exposed either to an atmosphere of acid vapours or to air charged with irritating particles of metal. The onset of the disease is sudden, with acute pains, followed by cyanosis and dyspnœa. Examination shows the presence of severe emphysema with crepitant râles and muffled breath sounds, but no bronchial breathing. The condition is very fatal, and the autopsy reveals throughout the lungs the greater part of the bronchioles obliterated and filled with an albuminous mass, the infundibula alone remaining free, and giving rise in consequence to the emphysematous state of the lungs. According to Fränkel both clinically and also post-mortem on macroscopic examination the condition may be mistaken for miliary tubercle, but in bronchiolitis obliterans the little solid nodules disseminated through the lungs are oblong or branched, instead of circular, as in the case of miliary tubercle. Little can be done therapeutically when once the disease is established. The patient succumbs in a state of intense dyspnœa, so that the chief indication is to utilise to the best advantage whatever lung tissue remains intact by the inhalation of pure oxygen gas.

In continuation of the discussion on "alimentary fever" at the recent meeting of the Society of Medicine at Berlin, to which reference has already been made in these columns, Dr. L. F. Meyer gave the results of his experiments with salt solution on infants. The objects of this research were to determine whether the rise of temperature occasioned by injections of saline was due to the quantity of salt or the concentration of the solution, whether the reaction depended upon the health of the infant, and whether the reaction was peculiar to sodium chloride or could be obtained by other salts of the same base or by different salts altogether. One hundred cubic centimetres of a 3-per-cent. sodium chloride solution were administered in two equal doses, with an interval of two hours, to infants under three months. The reaction consisted generally in a variable rise of temperature, which attained 40° C. six hours later and returned to the normal at the end of twenty-four to forty-eight hours. Healthy and dyspeptic infants gave the same reaction. With a physiological solution of 0.65 per cent. sodium chloride no effect was produced till the dose amounted to 300 cubic centimetres, and then only with dyspeptic infants. More definite results were obtained with slightly hypertonic solutions. All other salts except the sodium salts of the halogens failed to give any fever reaction. Dr. Meyer further experimented whether other salts, such as potassium or calcium chloride, might neutralise the effect of the sodium salt, but without any positive result. According to Dr. Meyer, therefore, this "saline fever" is determined by the sodium ions in presence of the halogen ions, and is in some way dependent upon an upset in the general metabolism. It might be suggested, however, that the sodium chloride acts directly upon the heat centre in the pons.

Two extraordinary cases of "pharmacomania" are recorded in an Italian medical journal by Dr. Zannini. The first case was that of a journalist of fifty-six years of age. At the age of forty he suffered from a severe gastro-enteritis, and he then commenced to take sulphate of magnesium periodically. At first he took a teaspoonful every other day, but this dose was soon insufficient, and he took it every day, then two and three teaspoonfuls every day. For five years the dose remained at about 50 grammes (nearly 2 oz.) every day, and was then increased to 70, 90, and 130 grammes (over 4 oz.) a day. It was then combined with magnesium carbonate and sodium bicarbonate. It is estimated approximately that within a year the patient absorbed about 25 kilos. of magnesium sulphate, 3 kilos. of carbonate of magnesium, and 5 kilos. of sodium bicarbonate. In spite of this abuse of the alkaline salts the patient showed no signs of the "alkaline cachexia" described by Nothnagel. He is capable of considerable work and fatigue, has a good appetite and no diarrhœa. The urine is normal in all respects, and most curiously remains strongly acid. In the second case a woman of sixty years some sixteen years previously commenced to use castor oil to correct severe constipation. Two teaspoonfuls from time to time were sufficient at first to produce the desired effect. Gradually the necessity became daily and the dose was also increased. Then the patient found that the castor oil acted as an appetiser and digestive. After meals she experienced a feeling of weight and discomfort in the stomach, which was removed by a dose of castor oil. In the morning, too, she experienced a feeling of discomfort till she had taken a dose, and altogether in this way she absorbed 76 grammes (about 2½ oz.) a day. She showed no sign of intestinal irritation or other trouble, and remained well nourished and even plump.

---

The following method of treating boils is recommended by Gallois: The smaller, imperfectly developed boils, and the larger ones if not ruptured, are painted with a solution of iodine in acetone (2 parts in 10). None of the lesions is incised. Those that have already ruptured are not painted. A piece of absorbent wool or a piece of lint, large enough to cover the area affected, is wrung out in glycerine or boracic acid (1 in 10). If wool be used, it must first be moistened in boiled water. The glycerinated dressing is applied and covered completely with a large wad of non-absorbent wool, which is then fixed firmly with bandages. The dressing is changed once or twice a day, according to the amount of pus discharged. If the suppuration is not abundant, the glycerine absorbs the whole of its moisture, and there is found only a yellowish staining of the wool. If the pus is a little more abundant, a soft, slightly adherent crust is formed. With a good deal of pus there may be some moisture over the surface of the boil, but not beyond. Under this dressing the lesions become rapidly less turgescent and the neighbouring skin is quickly freed from all miliary pustules, such as may have been produced under a previous dressing. Although some of the boils which have been painted with iodine may develop and discharge, they never become so voluminous, and no fresh ones are formed. Great care must be taken to disinfect the clothing, or reinfection may take place after apparent cure.

---

Resection of the sternum and affections of that bone in general receive but scant notice in the standard text-books; no doubt because of the rarity with which pathological processes in the bone are met with in this country. From Shanghai, however, Dr. W. H. Jefferys reports in the *China Medical Journal* four cases of necrosis of the sternum, one of the sterno-clavicular articulation, and one of superficial necrosis of the manubrium, all treated within five years. In two of these patients it became necessary to remove the manubrium and the whole sternum respectively for widespread necrosis with underlying abscesses. By these extensive operations the mediastina were so extensively laid bare that the ascending and transverse aorta, the innominate and common cartoid arteries, auricle, and portions of lung were exposed to full view, as shown by photographs taken at the time of the first dressing. It seems that there is no difficulty in the performance of the operation, and no more collapse of the chest takes place than during an extensive resection of ribs for chronic pleurisy. As to the origin of the condition Dr. Jefferys is still in doubt. In one case a cavity as large as an apple was opened in the lung; but in neither case was the tubercle bacillus to be found in the sputum, though both patients had cough and expectoration. One patient had a definite history of injury from a succession of heavy blows on the part; and it is suggested as a possible explanation of the other cases that the peculiar long-handled and heavy agricultural tools used by Chinese farmers may be wielded in such manner as to press upon and repeatedly to bruise the bone.

---

Dr. Cadeac recently read a paper on mercurialism before the National Society of Medicine of Lyons. His experiments were carried out chiefly on dogs and calves, and from them he was able to furnish the Society with several interesting observations upon the effects produced by mercury given in doses sufficient to produce toxic effects. One of the earliest symptoms of mercurialism is the appearance of stomatitis, but the author points out that there is no real relation between this symptom and the degree of intoxication. The stomatitis is a transitory phenomenon which, after being cured, will not reappear, however much of the drug be given. Simple irritants such as ammonia produce ulceration of the mouth by actual contact. Mercury, however, produces stomatitis indirectly. The resistance of the buccal tissues to bacterial invasion is lowered at first by the ingestion of the drug, with the result that the flora of the oral cavity proliferate unchecked and cause stomatitis. This lowering is, however, quite transitory, a sort of negative phase, and is followed by an increase in the resisting powers of the tissues. It is, therefore, evident that there can be no relation between stomatitis and the degree of mercurial intoxication. Moreover, the action of the mercury is not confined to the mouth, for the whole organism is affected in the same way, being at first deprived of its normal resisting powers to organisms and subsequently having these powers greatly increased.

---

The modern treatment of the malignant diseases of the uterus is practically a product of the twentieth century. Its advantages over the older methods, as well as its limitations and shortcomings, are well illustrated in a paper by Mrs. Garrett Anderson and Miss Platt in the *Journal of Obstetrics and Gynæcology of the British Empire*. There is, as the authors point out, little that is new disclosed by their study of 265 consecutive cases of these diseases; but there is confirmation of modern teaching and practice which is highly encouraging. It is pointed out that abdominal hysterectomy for cancer of the cervix and uterus has only recently superseded vaginal hysterectomy (and even less complete removals) as the routine operation. At the New Hospital for Women, in which institution the patients under consideration were treated between 1895 and 1907, there were up to the end of 1901 only two abdominal hysterectomies for cancer, whereas since then there have been but two vaginal hysterectomies. The authors claim also, probably quite correctly, that this hospital was amongst the first in Britain to adopt the newer operation. The primary mortality of vaginal hysterectomy at the hands of the surgeons to the New Hospital is 7.3 per cent.; that of abdominal hysterectomy 6.6 per cent. It is to be remembered that cases which would have been regarded as hopeless before the adoption of the abdominal route are now undertaken, and that very much more extensive removals are performed; so that the actual diminution in primary mortality is a greater triumph than the bare figures would appear to show. Glands are sought for systematically whenever the condition of the patient permits, and panhysterectomy with as wide a removal of the upper part of the vagina and the broad ligaments as is possible has been practised as a matter of routine for some years by all the surgeons.

---

The comparison of the primary mortality tells in favour of the abdominal route; but that of the after results amounts to demonstration of the same fact. Of patients suffering from cancer of the cervix, but one out of 29 is known to be alive and well after vaginal hysterectomy: in 12 the disease recurred rapidly, in two more within two years, and the remainder cannot be traced. Of 58 who have undergone abdominal hysterectomy, 26 are known to be alive and well at intervals of from 1½ to 4 years after operation; 17 have suffered from recurrence, 11 within a year, and the other 6 within 3½ years of operation; and 15 cannot be traced. Of carcinoma of the fundus uteri the picture is much less pathetic: 22 out of 39 are in perfect health at intervals of 2 to 7 years after operation, 7 have had recurrence, 3 are dead of other causes, and 7 cannot be traced. The well-known relations of fertility and cervical cancer, and of sterility and cancer of the fundus, together with the different age incidence of the two conditions, are well brought out in this series of cases. In 215 cervix cases the average age of the patients was 44.5 years, and the average number of children per patient was 5: only 6 of the women were unmarried and childless. In the fundus cases the average age was 58 years, the average number of children was one, and half the patients were childless altogether. The last and one of the most important facts disclosed by the investigation is that in every single case of cancer of the cervix pain was the last symptom of which complaint was made. In cancer of the fundus the first symptom was discharge in 17 cases, hæmorrhage in 10, and pain in 2. These results bear out all recent work on this important subject: they are worthy of the closest attention from both gynæcologists and general practitioners.

Teleman, writing in the *Deutsche Medizinische Wochenschrift*, describes a method for facilitating the discovery of the eggs of parasites in fæces. Five different samples, each about the size of a pea, are taken from the stool which it is desired to examine. These are placed together in a test-tube containing a mixture of equal parts of ether and hydrochloric acid. The tube is then shaken vigorously in order to break up the fæcal samples, gas being given off in the process. Neutral fats and free fatty acids are dissolved by the ether, and in the hydrochloric acid are found the albuminoid substances mucin, soaps, phosphates, and salts of lime. The large particles not dissolved are separated off from the mixture by means of a filter, the resulting filtrate being next centrifugalised for a minute. The filtrate will then be seen to consist of three layers. The topmost layer consists of the ether and the dissolved fatty bodies; the middle layer contains the hydrochloric acid, and the bottom layer is made up of substances insoluble in both reagents, such as cellulose, muscle fibres, and the eggs of parasites. These last can be seen with extraordinary clearness, even under a low-power microscope, after removing the supernatant reagents, but, of course, the number to be seen in any one field depends both on the number present in the stools and the quantity of fæcal matter examined.

---

Jochmann and Baetzner, in the *Munchener Medizinische Wochenschrift*, give an account of the therapeutic effects produced in surgical tuberculosis by a sterilised solution of 1 gram of trypsin in 100 grams of physiological serum. The fluid is injected directly into the affected tissues when there is no external opening, and in cases in which there are external lesions the injection is made into the peripheral tissues. Where there is much ulceration the sores are irrigated with the fluid, and covered with sterilised dry gauze. The injections are repeated at intervals, and vary in dose from 1 to 2 c.c. each time. With the exception of a slight smarting, this treatment causes no local or general disturbance. Rapidity of cure depends on several factors, such as the locality of the lesion and its previous duration, but the authors have found that tuberculous abscesses have healed up rapidly under its influence. However, the benefit is not confined to cases of abscess, for the primitive lesion, the source of the suppuration, undergoes a similar process of repair. Although the observations of the authors are numerous, they are too recent to allow of a definite opinion to be given as to the kind of cases in which the treatment can be used with success as well as to the permanency of the cures reported as having taken place. At present all that can be said is that pancreatic ferments are capable of digesting tuberculous tissues, while leaving healthy tissues unaffected, and at the same time inducing local hyperæmia and the energetic formation of granulation tissue. With the failure of trypsin treatment for cancer in mind, it is perhaps unwise to be too hopeful at present.

